# Phenological Changes in the Southern Hemisphere

**DOI:** 10.1371/journal.pone.0075514

**Published:** 2013-10-01

**Authors:** Lynda E. Chambers, Res Altwegg, Christophe Barbraud, Phoebe Barnard, Linda J. Beaumont, Robert J. M. Crawford, Joel M. Durant, Lesley Hughes, Marie R. Keatley, Matt Low, Patricia C. Morellato, Elvira S. Poloczanska, Valeria Ruoppolo, Ralph E. T. Vanstreels, Eric J. Woehler, Anton C. Wolfaardt

**Affiliations:** 1 Centre for Australian Weather and Climate Research, Melbourne, Victoria, Australia; 2 Kirstenbosch Research Centre, South African National Biodiversity Institute, Cape Town, South Africa; 3 CEBC, CNRS - UPR 1934, Villiers en Bois, France; 4 Department of Biological Sciences, Macquarie University, Sydney, New South Wales, Australia; 5 Department of Environmental Affairs and Tourism, Cape Town, South Africa; 6 Centre for Ecological and Evolutionary Synthesis, Department of Biosciences, University of Oslo, Oslo, Norway; 7 Department of Forest and Ecosystem Science, University of Melbourne, Creswick, Victoria, Australia; 8 Department of Ecology, Swedish University of Agricultural Sciences, Uppsala, Sweden; 9 Laboratorio de Fenologia, Departamento de Botânica, Instituto de Biociências, UNESP Universidade Estadual Paulista, São Paulo, Brazil; 10 Climate Adaptation Flagship, CSIRO Marine and Atmospheric Research, Brisbane, Queensland, Australia; 11 International Fund for Animal Welfare, Yarmouth Port, Massachusetts, United States of America; 12 Laboratory of Wildlife Comparative Pathology, Faculty of Veterinary Medicine, University of São Paulo, São Paulo, Brazil; 13 Institute for Marine and Antarctic Studies, University of Tasmania, Sandy Bay, Tasmania, Australia; 14 Joint Nature Conservation Committee of the UK, Stanley, Falkland Islands; 15 Animal Demography Unit, University of Cape Town, Rondebosch, South Africa; 16 Percy FitzPatrick Institute of African Ornithology, DST/NRF Centre of Excellence, University of Cape Town, Rondebosch, South Africa; Cirad, France

## Abstract

Current evidence of phenological responses to recent climate change is substantially biased towards northern hemisphere temperate regions. Given regional differences in climate change, shifts in phenology will not be uniform across the globe, and conclusions drawn from temperate systems in the northern hemisphere might not be applicable to other regions on the planet. We conduct the largest meta-analysis to date of phenological drivers and trends among southern hemisphere species, assessing 1208 long-term datasets from 89 studies on 347 species. Data were mostly from Australasia (Australia and New Zealand), South America and the Antarctic/subantarctic, and focused primarily on plants and birds. This meta-analysis shows an advance in the timing of spring events (with a strong Australian data bias), although substantial differences in trends were apparent among taxonomic groups and regions. When only statistically significant trends were considered, 82% of terrestrial datasets and 42% of marine datasets demonstrated an advance in phenology. Temperature was most frequently identified as the primary driver of phenological changes; however, in many studies it was the only climate variable considered. When precipitation was examined, it often played a key role but, in contrast with temperature, the direction of phenological shifts in response to precipitation variation was difficult to predict *a priori*. We discuss how phenological information can inform the adaptive capacity of species, their resilience, and constraints on autonomous adaptation. We also highlight serious weaknesses in past and current data collection and analyses at large regional scales (with very few studies in the tropics or from Africa) and dramatic taxonomic biases. If accurate predictions regarding the general effects of climate change on the biology of organisms are to be made, data collection policies focussing on targeting data-deficient regions and taxa need to be financially and logistically supported.

## Introduction

The relationship between the timing of life-cycle events and seasonal climatic patterns (i.e. phenology) is a fundamental biological process in both natural and managed systems. Phenology is a major driver in determining population dynamics, species interactions, animal movement and the evolution of life histories [1], [2]. Population-limiting factors are closely linked to seasonal or interannual phenological events, and shifts in phenology can affect ecosystems through changes in ecological interactions such as predator-prey and plant-pollinator dynamics [3]–[6] and the epidemiology of infectious diseases [7], [8]. Warming is hypothesised to lead to earlier spring events such as breeding onset, timing of flowering, breeding migration; delayed autumn events such as leaf fall, non-breeding migration; and a longer summer growing season [9]. Changing phenologies will contribute to shifts in species distributions, population viability and reproductive successes [10], [11] and in turn will affect climate via biogeochemical processes and the physical properties of the biosphere [12]. As such, phenological changes will have profound consequences for human societies and economies, including agricultural production [13], fisheries production [14] and human health [15].

Estimates for advancement of spring phenological events from meta-analyses range from 2.8±0.35 days/decade on land [9], [16], [17] to 4.4±0.7 days/decade in the oceans [18]. Substantial differences in the strengths of responses have been observed across taxonomic groups; datasets demonstrating significant trends showed that spring phenological events of herbs and grasses have advanced 1.1±0.16 days/decade, compared with 7.6±3.09 days/decade for amphibians and 3.7±0.7 days/decade for birds and 6.3±1.6 days/decade for marine phytoplankton [9], [18]. However, these meta-analyses are presently biased heavily to northern hemisphere temperate regions.

Since the beginning of the 20^th^ century the northern hemisphere has warmed faster than the southern hemisphere, with the rates of warming three times higher over land than ocean [19]. Further, comparisons of the magnitude of projected changes in temperature and precipitation, relative to the 20^th^ century mean and variability, consistently identify several low latitude and southern hemisphere locations as being amongst the first to experience novel or extreme conditions in the 21^st^ century [20], [21]. These include parts of the Amazon, Indonesia, southern Africa and Madagascar.

Given regional differences in climate change, and the disparities in projected climate changes between the northern and southern hemispheres (e.g. [22], [23]), it is likely that phenological shifts will not be uniform across the globe and that conclusions drawn from northern hemisphere temperate terrestrial and marine systems might not always apply to other regions, particularly so in the southern hemisphere. More than 80% of the southern hemisphere is ocean, which stores more heat than land and promotes different weather systems compared to the land-dominated northern hemisphere. For example, temperature variations are smaller and there is less extreme seasonal variation in the southern hemisphere, allowing some species to reproduce year round, particularly at lower latitudes [24], [25]. Instead of a clear period of biological dormancy, such as that enforced by low winter temperature at high northern latitudes, phenological cycles in the south are often less distinct. For example, in the high latitude oceans, low winter temperatures, sea ice extent and day length interact to produce prominent spring blooms of phytoplankton in the northern hemisphere, whereas blooms are less pronounced and of smaller magnitude in the southern hemisphere [26]. This means that continuous monitoring in the southern hemisphere may be more critical, and sophisticated statistical tools for interpretation will be required (e.g. [27], [28]). We might also predict the phenologies of species living outside European and North American temperate regions to show relatively less response to temperature fluctuations, but more to factors such as rainfall. For example, in many tropical systems where the alternation between dry and wet seasons is pronounced, the intensity and length of drier periods may drive the start of flowering and leafing seasons [24].

Given the climatic and biological differences of the two hemispheres and the paucity of southern hemisphere examples in previous phenological meta-analyses, our study aimed to collate all published accounts of recent phenological trends reported for southern hemisphere terrestrial, freshwater and marine species. Specifically, we sought to:

1. Determine the occurrence and magnitude of phenological changes among different regions of the southern hemisphere, and compare responses across taxa, marine versus terrestrial species, and seasons;
2. Identify climatic drivers of phenological change, and assess the relative roles of climatic versus non-climatic drivers;
3. Highlight data sets with >10 years of data that could be important for assessing future phenological responses; and
4. Identify major data and knowledge gaps, and suggest how these can be addressed to spur future research investigations.

Finally, we discuss how phenological information can inform our knowledge of the adaptive capacity of species, their resilience, and constraints on autonomous adaptation.

## Methods

Publications containing long-term phenological datasets were searched using the ISI Web of Science database and Google Scholar with keywords comprising: ‘phenology’, ‘warming’ and regional terms such as ‘Southern Hemisphere’, ‘Africa’, ‘Antarctic’, ‘Australia’, ‘South America’ or ‘New Zealand’. A candidate list of papers was checked for evidence of datasets or analyses that fitted our criteria (see below). Additional papers were added based on expert knowledge and through direct contact with specialists and researchers working with biological data and considering recent phenological reviews [24], [29], [30].

Studies were included in the database if they had at least 10 years of data and ended post-1990, thereby being coincident with the most recent climate changes. Data meeting the criteria were available from 1852 to 2011 (mean = 32 years, median = 25 years) with the bulk of the data from the 1970s–2000s. Long-term data sets ending before 1990 were also noted (but not analysed) because they are likely to provide useful baseline data for future comparative studies (see Appendix S1 in Supporting Information). The potential influence(s) of the lengths of data series and the starting years on the trends detected and conclusions is discussed in Appendix S4.

From each study, we extracted the following information for each species: study location, years of the study, species name, phenophases (e.g. breeding, migration, flowering) recorded, phenological trend (in days/decade), assessed or inferred climatic or non-climatic drivers of phenological variability or change (where available) and statistical significance (where available). Each phenological observation was classified as earlier, later or no change (trend not statistically significant).

In addition to the analyses presented in Tables 1, 2, and 3, which were based on all available data (up to 1208 time series), we formally analysed mean trends across time series which reported the trend together with an estimate of precision (standard error) using meta-analysis. Unfortunately, this information was reported for only 397 of the time series, of which 390 were from Australia. We therefore restricted the formal meta-analysis to Australian time series. Of these 390 times series, 164 measured trends in the reproductive phenology of the common grapevine, *Vitis vinifera* (119 trends were for harvest, 45 for maturity and three for flowering) and three for other plant species. For comparison, based on the full data set, information on grape phenology consisted of 176 of a total of 480 plant time series (see Appendix S5). A total of 223 of the 390 times series measured trends in breeding phenology of birds, which were more taxonomically diverse, covering 66 species. Variation thus enters this data set at three levels: among-species (within plants and birds, respectively), among-studies within species, and the error associated with estimating each trend. To account for this hierarchical structure, we adopted a hierarchical Bayesian approach similar to that used by McCarthy and Masters [31] to estimate the mean trend in phenology in birds and plants, and to quantify the uncertainty around these mean estimates. The model was a mixed-effects model with estimated trends as the response. Instead of treating these estimates as fixed data, we treated them as coming from a normal distribution with the mean and variance as reported in the original publications. Taxon (bird vs plant) was a fixed effect and species was treated as a random effect. The length of the time series used in this analysis varied from 10 to 115 years (median 27) and we included the logarithm of time series length as a further covariate in the analysis, as the length of the time series can influence the resultant trend estimate (e.g. [32], but see Appendix S4). WinBUGS code and additional detail is provided in the electronic supplement (see Appendix S2).

**Table 1:** Summary of southern hemisphere phenological data by region.

			*Trend over time (% trends)*				*Taxon*					*Habitat*		
Region	*N*	N*	*Earlier*	*Later*	*No Change*	*Mean* ± se *[range^§^]*	*Plant*	*Bird*	*Mammal*	*Arthropod*	*Other*	*Freshwater^†^*	*Marine*	*Terrestrial*
Antarctic/subantarctic*	50	31	7 (23%)	7(23%)	17(55%)	−6.7±6.3[−32.0, 18.7]	0	47	2	0	1	0	50	0
Australia/New Zealand	962	898	229 (25%)	70 (8%)	599(67%)	−4.2±0.6[−31.3, 22.9]	306	492	0	161	3	41	56	865
Africa	22	15	7 (47%)	3 (20%)	5(33%)	−1.2±1.5[−9.6, 10.8]	4	18	0	0	0	0	8	14
South America	173	1	0	1	0	–	170	1	2	0	0	0	3	170
Pacific nations	1	0	0	0	0	–	0	1	0	0	0	0	0	1
**TOTAL**	**1208**	**945**	**243 (26%)**	**81 (9%)**	**621(66%)**	−**4.2±0.6[**−**31.1, 22.7]**	**480**	**559**	**4**	**161**	**4**	**41**	**117**	**1050**

N is the number of datasets with a span of at least 10 years of data; 1208 data sets in total. N* is the number of datasets where trends over time [days/decade] were assessed – the three columns (earlier, later and no change [i.e. trend was calculated but was not considered statistical significant]; confidence level as reported in original papers, generally 5% level) sum to N*. Notes: * subantarctic regions under the jurisdiction of South America, Africa and Australia are included in Antarctic/subantarctic (e.g. Marion Island, Falkland Islands, Macquarie Island). ^†^ Freshwater species comprise Ardeidae (bitterns, herons and egrets), Anatidae (ducks and geese), Podicipedidea (grebes), Anhingidae (darters), and Phalacrocoracidae (cormorants). Marine species comprise penguins, seals, terns, gulls, albatrosses, petrels and shearwaters. ^§^ Range is based on 5^th^ to 95^th^ percentiles.

**Table 2:** Southern hemisphere phenological data set summaries.

(a)																											
	Taxon														Main foraging/growing habitat												
Trend over time (% trends)	Plant		Bird			Mammal			Arthropod				Reptile		Marine						Freshwater			Terrestrial			
Earlier	126 (45%)		108 (22%)			0			7 (4%)				2 (67%)		15 (21%)						6 (15%)			222 (26%)			
Later	6 (2%)		67 (13%)			0			8 (5%)				0		21 (30%)						11 (27%)			49 (6%)			
No Change	146 (53%)		326 (65%)			1			146 (91%)				1 (33%)		34 (49%)						23 (58%)			564 (68%)			
Mean ± se	−11.3±0.8		−1.1±0.8			–			2.3±1.7				−1.7±4.6		−4.9±2.9						−1.7±3.6			−4.3±0.6			
Range*^§^*	[−30.7, −8.2]		[−29.9, 27.7]						[−12.0, 16.7]				[−12.4, 9.0]		[−30.5, 20.7]						[−38.8, 35.4]			[−30.5,32.7]			
Ratio (−/+)	202:14		244:187			–			11:16				1:1		21:7						24:15			413:196			
**(b)**																											
	**Overall**								**Plants**									**Birds**									
**Trend over time (% trends)**	**Autumn**		**Winter**	**Spring**			**Summer**			**Autumn**	**Winter**			**Spring**			**Summer**		**Autumn**			**Winter**			**Spring**		**Summer**
Earlier	39		22	30			25			62	0			31			52		17			22			30		15
No Change	51		67	60			64			38	0			62			48		64			67			60		69
Later	10		12	11			12			0	0			7			0		19			12			10		16
N	213		51	225			85			105	0			29			21		108			51			192		62
Ratio (−/+)	142: 65		32:15	128:57			42:23			102:3	0:0			19:7			17:0		40:62			32:15			107:49		25:22
**(c)**																											
**Trend over time (% Trends)**		**Breeding**			**Migration**			**Emergence**				**Flowering**				**Maturity**				**Harvest**			**Pollen/Fruiting/Seeding**				
Earlier		27 (20%)			83 (22%)			7 (4%)				23 (22%)				24 (53%)				78 (66%)			0				
Later		24 (18%)			44 (12%)			7 (4%)				6 (6%)				0				0			0				
No Change		83 (62%)			244 (66%)			146 (92%)				81 (72%)				21 (47%)				41 (34%)			3 (100%)				
Mean ± se		−0.4±1.0			−1.3±1.1			2.3±1.7				−5.6±2.8				−14.0±0.9				−12.7±0.7			−7.8±5.5				
Range*^§^*		[−16.3, 15.4]			[−32.9, 30.4]			[−12.3, 16.9]				[−37.8, 26.6]				[−23.9, −4.2]				[−26.1, 0.6]			[−23.4, 7.9]				
**Total**		**174**			**385**			**160**				**209**				**57**				**119**			**94**				
Ratio (−/+)		54:44			191:145			11:15				39:10				45:0				115:3			2:1				

(a) Number of southern hemisphere phenological data sets by taxon and main foraging habitat, (b) Summary of direction of trends in southern hemisphere phenological data (%) by main season of phenological event, as a percentage of cases.

(c) Summary of southern hemisphere phenological data (number) by phenophase.

Not all datasets had published trends (and those that did were predominantly from Australia, see text for details) or directions of change and only those which explicitly tested for temporal trends are included here. A subset of these, which also recorded the standard error of the trend estimate, is analysed in more detail in Appendix S2. No change indicates a trend was calculated but was not considered statistical significant (confidence level as reported in original papers, generally 5% level). Mean trend in days per decade. ^§^ Range is based on 5^th^ to 95^th^ percentiles. Ratio (−/+) is the ratio of the number of negative to the number of positive trends observed, irrespective of the significance of the trend. Not all studies provided trends estimates [e.g. days/year] so the sum of the two ratio values do not equal the sum of Earlier, Later, No Change (Table 2a), N in Table 2b or the sum of the two ratio values. South American plant datasets were classified as wet or dry season but, as none had trends recorded, they have been excluded from this table.

**Table 3:** Summary of identified climate drivers of phenological change in the southern hemisphere.

		Main foraging habitat			Region			Trend Direction			
Climate driver	Total	Marine	Freshwater	Terrestrial	Africa	Antarctic/Subantarctic	Australasia	South America	Earlier	Later	No Change
ENSO/SOI	**104**	13 (11%)	0	91 (9%)	3 (14%)	6 (12%)	13 (1%)	82 (48%)	3	4	7
SAM	**2**	2 (2%)	0	0	0	2 (4%)	0	0	1	0	1
SST/Sea level	**16**	16 (14%)	0	0	1 (5%)	4 (8%)	11 (1%)	0	2	7	3
Sea ice extent	**15**	15 (13%)	0	0	0	15 (30%)	0	0	1	6	8
Snowmelt	**8**	0	0	8 (1%)	0	0	8 (1%)	0	3	1	2
Air Temperature	**474**	12 (10%)	8 (20%)	454 (43%)	4 (18%)	5 (10%)	381 (40%)	84 (49%)	146	18	207
Rainfall/No. Rain days	**296**	3 (3%)	18 (44%)	275 (26%)	5 (23%)	0	125 (13%)	166 (97%)	23	20	85
Sunshine hours	**3**	0	0	3 (<1%)	0	0	2 (<1%)	0	–	–	–
Wind	**1**	1 (1%)	0	0	0	1 (2%)	0	0	0	0	1
None identified	**140**	5 (4%)	17 (41%)	118 (11%)	0	0	140 (15%)	0	39	9	92
Climate not tested	**419**	64 (55%)	3 (7%)	352 (34%)	14 (63%)	23 (46%)	377 (39%)	5 (3%)	44 (18%)	29 (35%)	281 (45%)
**Total**	**649**	**48**	**21**	**580**	**8**	**27**	**546**	**168**	**159**	**44**	**248**

‘None identified’ refers to studies that explicitly examined relationships between the climate variables considered in that study and phenology but found no statistical significant relationships. Note some studies identified more than one climate driver. Total is the number of datasets that were considered to have at least one climate driver for phenological variability or change. No change indicates a trend was calculated but was not considered statistical significant (confidence level as reported in original papers, generally 5% level).

## Results

### Collated Studies of Phenological Trends Amongst Southern Hemisphere Species

We identified a total of 1208 long-term southern hemisphere phenological time series from 89 studies (Appendix S3), on 347 species. The majority of datasets were from three regions: Australia/New Zealand, South America and the Antarctic/subantarctic (Table 1); relatively few came from studies undertaken in the tropics or from Africa.

Studies primarily focused on plants and birds, particularly seabirds, and were predominantly from terrestrial species, with fewer observations of marine and freshwater species (1050, 117 and 41 time series respectively, Table 1; Appendix S5). Under-represented taxa comprised amphibians, reptiles, mammals, fish and invertebrates. The most commonly recorded phenophases were breeding and migration for birds, emergence date for arthropods, and flowering, fruiting (including pollen and seeding), maturation and harvest dates for plants (Table 2c). Records for reptiles (n = 3) were only of breeding timing in Australia.

### Occurrence and Magnitude of Phenological Changes Amongst Southern Hemisphere Species

Overall, southern hemisphere phenology advanced by an average of 4.2±0.6 days/decade (n = 606; median = −4.0 days/decade; Table 1a), higher than the estimated advance for northern hemisphere terrestrial species (2.8±0.35 days/decade; [9]) but comparable to the advance for predominately northern hemisphere marine species (4.4±0.7 days/decade; [18]).

Of the 1208 time series considered, 78% assessed the statistical significance of observed trends (Table 1). No statistically-significant temporal changes in phenology were identified for the majority of time series (66%). However, when significant changes over time were noted (p<0.05), the timing of events was more likely to shift earlier than later (26% versus 9%, Table 1); the mean rate of advance for events was 14.4±0.7 days/decade (5^th^ and 95^th^ percentiles: −31.6 and −1.6) and later events delayed by 20.4±2.2 days/decade (5^th^ and 95^th^ percentiles: 2.6 and 49.5). There was no effect of the length of the data series or the year in which the data series commenced on the likelihood of detecting an earlier or later trend over time (Appendix S4). However, the length of the data series clearly influenced the magnitude of the trend observed (Appendix S4), with the start year having only a marginal effect. In general, more pronounced trends were observed for shorter time series and in those series starting in more recent years.

#### Regional comparisons

There appeared to be regional differences in the direction and magnitude of the trends observed, although these were sometimes based on a low number of studies (Table 1). For example, there was a near-even split between earlier and later events in the Antarctic/subantarctic, where sea-ice extent has a strong influence on primary production and phenological events (e.g. [33]]. The trend towards earlier events was more evident in Australian and the African region (mean rate of change −4.2±0.6 and −1.2±1.5 days/decade, respectively).

#### Taxonomic comparisons

Across taxa, terrestrial plants (most of which were from temperate regions; 63% from Australian grape vines) demonstrated the strongest signal with 45% of time series reporting a statistically significant advance in phenology, compared with just 2% demonstrating a significant delay (Table 2a). The mean rate of change across all plant phenophases was −11.3±0.8 days/decade (5.6±2.8, 14.0±0.9 and 12.7±0.7 days/decade earlier for the flowering, maturity and harvest datasets, respectively; Table 2c), with no trends towards later events observed among the maturity and harvest datasets (all maturity and harvest time series are from vineyards in southern Australia). Flowering events were generally spring events, and maturity and harvest events occurred during summer or autumn. These advances are faster than the reported rates for spring events in land plants from the northern hemisphere (1.1±0.2 to 3.3±0.9 days/decade; [9]).

Among birds, 35% of time series reported a statistically significant trend (advances = 22%; delays = 13%), with a mean rate of change of −1.1±0.8 days/decade. Spring events (which include departure for migrations) advanced by 2.2±1.4 days/decade, slower than the spring advances for northern hemisphere birds (3.7±0.7 days/decade; [9]). The climate and physical geography of the southern hemisphere is associated with higher proportions of nomadic or partially migrant birds [34], [35] with variable breeding and migration schedules that may not be tied as closely to spring timing as in the northern hemisphere temperate regions.

The meta-analysis of Australian records (n = 390) found a mean advancement for plants of 9.7 days/decade (95% credible interval 12.1 to 7.3, Appendix S2) and for birds 2.6 days/decade (3.7 to 1.6), making the difference in the mean trends between the two groups 7.1 days/decade (4.5 to 9.7). This, more formal analysis, supports the results of the full data set presented above (Table 2a); that southern hemisphere plant phenology is advancing at a much greater rate than that of birds.

Few statistically significant trends over time were observed for arthropods (Table 2a), and they were almost equally likely to be earlier as later, although the number of observations is low and restricted to one taxonomic group: butterflies. The magnitude of significant trends for butterflies was lower than for plants (earlier events: −7.3±1.8 [n = 7] and −14.8±0.7 [n = 124] days/decade, respectively; later events: 9.7±2.3 [n = 8] and 19.1±3.4 [n = 6] days/decade, respectively).

For species with significant phenological shifts, those inhabiting freshwater or marine systems were significantly more likely to demonstrate delays than terrestrial species (Maximum Likelihood Ratio chi-square 82.7, df = 4, p<0.001; Table 2a).

#### Seasonal comparisons

The season in which the phenophase occurred was not related to the direction of trend over time (Maximum Likelihood Ratio chi-square 13.5, df = 8, p = 0.097). Although significantly earlier trends were more often observed than later ones for all seasons, the greatest proportion occurred during the autumn (March - April, Table 2b). The trend towards earlier events in autumn, compared to the other seasons, was driven largely by observations on plants (particularly Australian grape vines). Trends in autumn and summer avian phenologies were more evenly split between statistically significant advances and delays (Table 2b).

### Climatic Drivers of Phenological Change

In 789 of 1208 (65%) phenological data series, the researchers explicitly considered at least one climate variable as a potential driver of the observed phenological variability and/or change (Table 3; includes cases where a climate driver was identified and where climate variables were tested but not considered a driver). However, temperature was the only variable considered amongst 27% of papers. A low number of studies included potential non-climatic drivers, such as observational and management changes. For species foraging in marine environments, the most frequently identified drivers of phenological change were sea ice extent (in the Antarctic), broad-scale indices such as the El Niño-Southern Oscillation phenomenon (ENSO) and the Southern Oscillation Index (SOI), and sea surface temperature. For freshwater species, rainfall and air temperature were the most frequently identified drivers. Similarly, among terrestrial species, air temperature was cited as the primary driver of phenology in 52% of cases, whereas 26% found rainfall to be the primary driver. In contrast, 11% of studies did not identify a climate driver.

Regional differences were also evident in the relative importance of climate drivers (Table 3). For the Antarctic/subantarctic region, sea-ice extent was most often cited as the primary variable (30% of cases), with ENSO (12%) and sea surface temperature (8%), likely covariates, also playing an important role. In contrast, rainfall was reported as a primary driver in southern Africa (23% of cases) with air temperature and ENSO also noted as playing an important role (18% and 14% of cases, respectively). Similarly, for tropical South America rainfall was identified as the main driver, especially in seasonal environments, with day length also important in non-seasonal regions, and temperature becoming increasingly important at higher latitudes. Conversely, for Australasia, air temperature was more often considered the primary driver (40% of cases), followed by rainfall (13%).

Trends towards earlier phenological events were most often associated with increasing air temperatures (73% of all earlier events considered air temperature as a primary climate driver) (Table 3). In fact, 39% of datasets that considered air temperature as one of the primary drivers observed earlier phenologies, compared to 5% observing later phenologies. For datasets where rainfall was identified as the primary climate driver there was a near-even mix of earlier and later events. Where sea-ice extent was noted as a primary driver, a greater number of later events were observed than earlier ones (6 of 15 compared to 1 of 15, respectively). This was also the case for sea surface temperature/sea levels (2 of 16 earlier compared to 7 of 16 later).

## Discussion

Based on 1208 datasets from 347 species, this is the most comprehensive review of phenological trends and their associated drivers among southern hemisphere species. We show that a broad variety of taxa from terrestrial, freshwater and marine realms within the southern hemisphere have experienced changes in the timing of their life cycles in recent decades. The dominant patterns to emerge from our study, consistent with findings from the northern hemisphere [9], [16]–[18], [36], include a) dramatic biases in the regional availability of data and reported taxa, b) an advance in the timing of spring events (based predominantly on southern temperate species), c) an expectation of mismatches in the timing of key life history events between trophic levels, d) substantial differences in the magnitude of phenological changes between taxonomic groups and regions, and e) phenological changes are often correlated with temperature. We found in many cases, changes were faster than those reported from predominately northern hemisphere regions. We also found that precipitation frequently appears to play a key role and, in contrast with temperature, the direction of phenological shifts in response to altered precipitation regimes is difficult to predict *a priori*. This is due, in part, to greater certainty in direction of future change in temperature than precipitation [19], [37].

### Regional and Taxon-specific Biases in Data Availability

It is clear that serious data deficiencies exist in southern hemisphere phenological datasets, with continental-scale knowledge gaps in relating phenology to climate variables and change. This is particularly evident when comparing the number of available datasets for the three main land masses. Despite Australasia being a fraction of the size of Africa or South America, more than 80% of the available datasets originated there, as did almost all of the phenology trend analyses (Australia/New Zealand versus Africa/South America; 898 versus 16; Table 1). The situation for the Pacific nations, which are rich in island endemic species and may face unique challenges during climate change (e.g. extreme barriers to adaptive distribution shifts), is even more dramatic with only a single bird dataset found (Table 1).

When the taxa represented in these datasets are examined, extreme biases are also evident. Plants and birds comprise around 85% of datasets, arthropods 13%, and mammals, reptiles and amphibians each contributing less than 1%. But even these figures are misleading as to the nature of biases. Although 480 plant datasets were recorded, phenological trends were predominantly from Australia/New Zealand, only four from Africa and none from the other regions, and 60% of these of studies were on commercial viticulture crops. All 161 arthropod studies were from 68 species of Australian butterflies. The fact that there are only three studies on reptiles, four on marine mammals, and none on amphibians suggests that our predictive capacity of how phenology might change over time and in response to climate drivers for a huge range of taxa will essentially be guesswork for many years to come.

### Regional Differences in Species Responses, Timing of Events, and Climate Drivers

Environmental or climate change drivers vary regionally, and may result in different biological responses [37]. For example, large areas of cooling have been observed in the Southern Ocean during the past few decades, whereas West Antarctica and several subantarctic islands have warmed more rapidly than other parts of this continent (e.g. [38], [39]). Similarly, terrestrial biomes have warmed more rapidly than marine biomes [19]. Analysis of observed seasonal shifts in temperature over 1960–2009 showed high variability between hemispheres and within regions [40]. Because of the smaller seasonal thermal variations in the southern hemisphere, spring temperatures were recorded earlier by 2.5 days/decade over oceans and 2.2 days/decade over land, whereas in the northern hemisphere, the corresponding values are 2.1 and 1.5 days/decade, respectively. In many cases, changes in observed phenology are faster than or lag shifts in seasonal temperatures, suggesting the potential for decoupling of trophic and other interactions within ecosystems.

Phenological trends among populations of a widespread species may differ in magnitude, depending on local and regional changes in climate. This is demonstrated among populations of Dollarbirds *Eurystomus orientalis,* and Common Koels *Eudynamys scolopacea,* which have significantly advanced their arrival at breeding grounds in south-east Australia but not in more northerly regions [41]. Similarly in south-east Australia, the first flight date of inland populations of the butterfly, *Heteronympha penelope*, has been significantly delayed since 1950, while no significant trend has occurred amongst coastal populations [42]. In addition, several studies have demonstrated that fish populations located at the limits of their geographical distribution are more sensitive to environmental variability than core populations (e.g. [43]). This suggests that higher latitude species, which are often at their geographical and ecological limits, may respond more strongly to climate change.

Advances in the timing of breeding have been reported for seabirds in the Arctic due to increasing spring temperatures and earlier access to nesting sites and food availability [44], [45]. In Antarctica, however, studies report both delayed breeding among seabirds [33], [46], [47] and advances [46], [48]. However, both delays and advances are generally consistent with expectations under climate change when local and regional climatic and oceanographic changes together with seabird life histories are taken into account [33]. Marine food webs near Antarctica are heavily affected by variations in the extent of sea ice, which is in turn influenced by changes in salinity, sea temperature, winds, and ocean circulation [33], [49]. Increasing sea surface temperature and decreasing sea ice extent are associated with reductions in Antarctic Krill *Euphausia superba* and other marine organisms, and may be responsible for delayed breeding in seabirds [33], [50]. At a regional scale, if separate populations of migrating seabirds use different over-wintering areas, climatic differences between these areas during the pre-breeding stage may contribute to divergent trends in the timing of breeding, for example, through regional differences in food quality and peak availability. Similarly, individuals that occur (or breed) at the edge of the species’ geographical distribution, where they are likely to be at the limit of their physiological tolerance, may be more likely to undergo phenological adjustments (or indeed disperse) than individuals closer to the core of their distribution (e.g. Gentoo Penguins *Pygoscelis papua*, [51]). This could partly explain why climate change may affect Arctic and Antarctic species differently: the lower extent of land cover in the southern hemisphere may more tightly constrain the responses of populations, compared with those in the northern hemisphere.

Individuals and different species within the same biome or region may vary in their response to climate change because of the scale at which they sample their environments (e.g. through predation) and use environmental cues [52]. In the Southern Ocean, the crustacean genus *Euphausia* (krill) plays a critical role in the food web of the Scotia Sea, supporting many top predators [53], whereas in the Southern Indian Ocean the fish family Myctophidae dominates the food web [54]. Yet, there are top predator species common to both biomes. Consequently, this spatial variability at lower trophic levels coupled with specific temperature tolerances may result in variable impacts of regional warming, with different consequences for upper trophic levels depending upon the regional food webs in which they reside (e.g. [55]).

Differences in regional phenological responses may also result from variation in food webs and associated environmental drivers. Top predators (such as seabirds) integrate the effects of environmental or climatic change in the lower trophic levels over a range of temporal and spatial scales, and regional differences in food webs may mean that top predators are affected differently in different regions. Similarly, initiation of breeding, and other phenological parameters, may vary depending on the availability of resources before the breeding season. The laying season of Peregrine Falcons *Falco peregrinus,* near Canberra, Australia, for example, is extended during drier winters, and rainfall directly and indirectly influences the breeding of these predators [56]. More rainy days in the pre-laying period may reduce hunting efficiency or result in a chilling effect, reducing the bird’s energy stores, and influence the timing and subsequent success of breeding. Higher rainfall, in contrast, may shorten the egg-laying period if nests are flooded or damaged [56].

### Key Uncertainties in Climatic Drivers of Phenological Trends

A number of factors confound our ability to identify drivers of phenological change. First, most of the studies analysed in this review that attempted to relate phenology to specific drivers focused on one climate variable only, most commonly air or sea surface temperature. Mismatch among the temporal and spatial scales of biological and climatic datasets and lack of process-understanding can reduce ability to obtain reliable inferences [99]. The focus on temperature limits our understanding of the explanatory power of other variables, such as precipitation or sea ice extent.

Second, the scarcity of long-term datasets in some southern regions, in particular in Africa, Pacific nations and South America, combined with the potentially strong (but poorly understood) effects of ENSO may bias analyses towards identifying ENSO fluctuations as phenological drivers. Rainfall and temperature patterns in tropical and southern hemisphere regions are strongly influenced by ENSO, which occurs in pseudo-cycles of two to seven years [57]. As such, ENSO events have substantial impacts on ecosystems, and drive pulses of primary productivity in tropical, arid and semi-arid southern regions [58], [59]. Third, given that precipitation frequently drives species life cycles, focusing on a) temperature only, and b) identifying only linear trends in phenology may mean that important non-linear responses of populations to precipitation (and temperature) variability are missed.

Finally, phenological data sets are frequently from extensively modified and perturbed ecosystems. Less disturbed ecosystems may be more resilient [60], and analyses and predictions derived from them may be more robust. The preservation of the remaining minimally modified ecosystems is therefore critical, both for climate adaptation [61] and for analyses investigating impacts of climate change.

### Non-climatic Drivers of Change

Although non-climatic factors, such as phylogenetic constraints [62], [63] and competition [64], [65], can be important drivers of phenological trends, few studies identify or discuss the potential for non-climatic drivers to fully, or even partially, explain trends (but see [66]). Exceptions include the potential impacts of changed fishing practices on seabirds [67]; the role of UV radiation affecting Antarctic plants [68]; solar radiation on plants [68], [69] and changed agricultural practices [70] on earlier wine grape ripening. Several studies also describe the impacts of experimental banding on survivorship and breeding phenology of penguins [71]. Others suggest that reductions in the density of breeders, regardless of cause, may reduce social stimuli associated with breeding, with consequences for breeding phenology [72], [73]. Phenological advances in the timing of breeding by Wandering Albatrosses *Diomedea exulans* at Bird Island, South Georgia (South Atlantic Ocean) may also be driven by within-individual intrinsic change (ageing) rather than phenotypic plasticity [74].

### Adaptive Capacity of Southern Hemisphere Species

Populations can respond to environmental change in three main ways: evolutionary change, phenotypic plasticity *in situ*, and dispersal (changes in distribution). Empirical information on the evolution of phenotypic plasticity in the southern hemisphere is very scarce, highlighting the need for longitudinal and inter-specific comparative studies. For species with relatively long generation times, phenological changes through micro-evolutionary processes may be too slow to track the pace of projected climate change [75].

Phenological flexibility may provide an important buffer against negative impacts of environmental change, and a useful indicator for identifying species capable of adapting. The ability of Antarctic/subantarctic species to alter their phenology in response to climate variability and change is poorly known. However, many seabirds and marine mammals are heavily constrained in their phenology by the short time period at high latitudes with favourable conditions for breeding. For migratory seabirds in more southerly regions, the timing of breeding is likely to be determined by photoperiod modulated by other factors, such as sea surface temperatures, rainfall and food availability. Because photoperiod is not affected by climate change, it is not necessarily a reliable clue of peak/optimal breeding conditions.

Penguins are a taxon that dominates reports of phenological shifts in the Antarctic (e.g. [76]). Substantial differences occur among these species in their ability to adapt via phenological change. As an example, Adélie Penguins *P. adeliae* at the South Orkney Islands normally commence breeding earlier than Gentoo Penguins, except in years with mild winters when Gentoo Penguins breed first [77]. The more limited flexibility in the phenology of Adélie Penguins may result from other adaptations that enable the species to breed successfully at higher latitudes than Gentoo Penguins [77]. In addition, clutch initiation dates in the non-dispersive Gentoo Penguin have adjusted more rapidly than in the dispersive Adélie or Chinstrap Penguins *P. antarctica* [48], [51], [78]. However, data suggest that in general, penguins may have a limited ability to adapt phenologically to climate change, and have so far responded primarily via dispersal (e.g. [79], [80]).

Limited availability of suitable breeding grounds on oceanic islands, as well as high levels of philopatry, may reduce the ability of seabirds and marine mammals to respond via dispersal [77], [79]. Island species are often sedentary, with a reduced capacity for range shifts, and lower genetic variability that may limit evolutionary rescue effects if phenotypic plasticity cannot operate. These characteristics potentially make island species more vulnerable to rapid climate shifts (e.g. the Mauritius Kestrel *Falco punctatus* [81]). However, some highly migratory bird species, such as the South Polar Skua *Catharacta maccormicki* show a wide variation in their migration behaviours, even within a single breeding population, indicating the potential for buffering in some species [82].

Several studies from the northern hemisphere provide evidence that the tendency of species to alter their migration and breeding phenology over the past few decades is correlated with population trends [2], [83]. Examples of correlations between phenological responses and demography, however, are lacking in the southern hemisphere studies reviewed here, as are similar investigations of correlations between life history characteristics and phenological change. However, recently published studies from the northern hemisphere provide useful pointers for future investigations. Amongst passerines in France, for example, species with the broadest ecological and thermal niches, shortest mean migration distances and largest brains were most able to adjust their phenology, indicating that specialists and long distance migrants are more at risk [84]. The migration of Barn Swallows *Hirundo rustica* from southern Africa appears to be constrained by the need to complete their moult before leaving their non-breeding grounds [27], [85]. For highly specialised species, it would be useful to investigate whether individuals use environmental clues to identify optimal breeding times, or whether the timing of breeding is affected more by constraints before breeding.

### Mismatches between Trophic Levels

Southern plant phenology (i.e. flowering, maturity and harvest dates) has shown larger shifts towards earlier timing, in the available datasets, than other taxa. This has the potential to result in mismatches in the timing of life history events for dependent species (*sensu* [3], [86]). However, few studies provided data on changes in the phenological synchrony of interacting species, although several speculate as to the potential impacts of such changes and relationships. For plants and their seed predators in New Zealand, it is predicted that warming will eventually result in annual flowering of mast seeding species, with subsequent increases in insect and mammalian seed predation, changes in the population regulation of seed predators, and a decrease in predator satiation leading to decreasing reproductive output in the plants [87], [88]. Trophic mismatches are expected in Antarctic/subantarctic penguins (reviewed by [77]), because the return of penguins to breeding grounds is dictated by non-climatic cues such as photoperiod. With warming and earlier retreat of sea ice, shifts in prey phenology may lead to increasing asynchrony between some penguin species and their prey.

In general, observed phenological adjustments amongst seabirds and marine mammals have been small, and larger adjustments may rapidly create a temporal mismatch with requirements for raising offspring, resulting in depressed recruitment and population declines. Similarly, phenological flexibility among Antarctic fur seals *Arctocephalus gazella* is likely to be limited because females are constrained by the relatively long interval (∼16 months) between conception and weaning of their pup. This suggests that the loss of predictability of food supply is likely to affect long-term fitness [89].

Changes in Bogong moth *Agrostis infusa* phenology in the Australian alpine zone are also expected to impact on their vertebrate predators [90]. The moths now arrive significantly later than in the past, with potential negative impacts for birds and mammals that prey upon the moths in spring.

### Detection, Causality and Attribution

Challenges associated with attributing phenological changes to climate change include the complication of changing light regimes with latitudes, climate-driven shifts in the amplitude of seasonal climate cycles [91] and the potentially slow time to emergence (>50 years) of climate change signals in many regions [92]. Biological systems are complex and respond to climate at local or regional scales. Interactions among drivers are common, and attribution of individual biological change tends to be difficult and costly [93], [94].

However, there are many approaches to attributing detected phenological changes to recent climate change and other drivers; best practices involve partitioning responses among a set of causes or drivers of change [95], [96]. Meta-analyses documenting systematic biological changes across many species and regions, that are consistent with climate change occurring over the same period and linked to rising anthropogenic CO_2_ emissions, are one a form of attribution [93]. Using all southern hemisphere terrestrial datasets with a significant phenological trend (n = 271), we found that 82% showed a response in the direction consistent with anticipated responses to increasing temperatures (i.e. an advance in phenology). This is similar to that observed among northern hemisphere terrestrial datasets (87%, [16]). In contrast, whereas 75% of northern hemisphere marine datasets demonstrate a similar advance in phenology [18] as in the terrestrial realm, only 42% of those in southern hemisphere oceans do (Table 2a).

### Addressing Knowledge Gaps

Long-term biological data sets are a fundamental pre-requisite for regional, continental, hemispheric and global analyses of biological responses to climate change, and effective conservation interventions where needed. Reducing the spatial, temporal and taxonomic gaps in these data will improve confidence in the veracity of analyses, detection of trends and responses, and predictions arising from analyses. This paper clearly identifies many taxa (particularly mammals, reptiles, amphibians), ecosystems (especially freshwater) and regions (particularly Africa, small island nations, South America) for which our knowledge of phenological responses is still very limited. Important knowledge gaps also need to be addressed in the Antarctic/subantarctic region, where coverage of long-term datasets is mainly limited to birds (94%) and mammals (4%) and do not reflect the dominance of marine invertebrate and fish species. Recently, however, efforts to monitor inter-annual variations in spatial distribution, abundance and phenology of fish species playing a major role in the Antarctic marine food web, such as the Antarctic silverfish *Pleuragramma antarcticum,* have been initiated [97].

Quantifying or predicting shifts in phenology largely relies on long-term biological and climate data [16], [98], [99]. The observational bias between the northern and southern hemispheres has arisen partly due to the long histories of scientific investigation and academic institutes in many northern hemisphere countries [100], [101]. This is compounded by the trend for major funding institutions rewarding the *quantity* of published papers per year, thereby reducing the likelihood of researchers’ accumulating and synthesizing large datasets over the long term. However, renewed interest in citizen science and the development of online and mobile recording tools [102], [103] should facilitate enhanced scientific enquiries into biological trends in coming years for other parts of the globe.

In Australia, gaps and absences of long-term biological data are widely recognised (e.g. [104], [105]). Australia is conspicuous for the absence of long-term ecological research (LTER) initiatives analogous to those undertaken in Europe and North America, with a network only recently being established (http://tern.org.au/Long-Term-Ecological-Research-Network-pg17872.html). In South America, the consolidation of large LTER programs in Brazil (PELD - Programa de Pesquisa Ecológica de Longa Duração and SELD – Stios de Estudos de Longa Duração) will also provide valuable information on species’ responses to climatic change and other man-driven environmental disturbances (http://agencia.fapesp.br/16275; http://www.cnpq.br/web/guest/apresentacao7). Southern Africa has had LTER initiatives since the late 1990s, but most national networks are unfunded or poorly funded (e.g. [106]).

There are a number of long-term population datasets from southern hemisphere regions that have not yet been analysed (and published) such as plant datasets (n = 170) from South America (LPC Morellato pers. comm.) and we strongly recommend exploration of these at the earliest opportunity. Other important sources of information that could fill some of the gaps mentioned above are phenology data from herbarium and museum collections (e.g. [107], [108], [109]). Further, the establishment of regional phenological networks that facilitate data collection from a wide range of researchers and amateurs, such as has recently occurred in Australia (ClimateWatch, multiple species including both terrestrial and marine http://www.climatewatch.org.au) and New Zealand (national plant network http://nzpcn.org.nz/page.asp?flora_phenology), would be invaluable to future scientific inquiries. Other new initiatives promise to fill some existing knowledge gaps, including those focusing on traditional ecological/biological knowledge by the Climate and Oceans Support Program in the Pacific (www.bom.gov.au/COSPPac). International collaboration in data collection and exchange would likewise greatly facilitate further research, such as the Science Without Borders program in Brazil (http://www.cienciasemfronteiras.gov.br).

### Phenological Predictions

Our limited capacity to predict the magnitude and direction of climate change impacts on phenology constrains our ability to provide more than a few generalisations as to the relative vulnerability of species to future changes in climate. We present these below, and suggest relevant questions to be addressed by future research:

- Highly synchronised species (particularly with regards to breeding) are at greater risk of adverse impacts of climate change than are opportunist or generalist species. If synchrony is considered a continuous variable, is there a correlation between synchronicity and degree of impact?
- Highly specialised species (particularly with regards to dietary preferences) are at greater risk of adverse impacts of climate change than are opportunist or generalist species, primarily through the risk of predator-prey mismatches in time and/or in space. How do we determine prey specialization in predators, and can we relate the degree of specialization to prey abundance and availability?
- Migratory species (particularly trans-hemispheric) face cumulative pressures from climate change both at their breeding and non-breeding areas. Can we predict a correlation between the degree of adverse impacts on species that are considered resident, dispersive or migratory?
- Species with restricted distributions (e.g. oceanic and ‘climatic’ islands, altitudinal and latitudinal isolates) are at extreme risk of adverse impacts of climate change because they have fewer (or nil) options than do opportunist or generalist species. Are single-island endemics most at risk of adverse impacts?

### Data Caveats

Whilst we have identified a number of phenological datasets, and their associated trends, for southern hemisphere species, it is important to point out that analyses based on these datasets may be prone to biases and limitations which may influence results. For example, the datasets incorporated in this study differ in terms of observer effort and biases and the use of a variety of different measures of phenological timing, both of which are beyond the control of this review. The use of phenological measures based on first or last events has been argued to be less than ideal [110], particularly in relation to the potential influence of population size, outliers and observer effort and ability [111]. However, these were the most common observations recorded (Appendix S6), most likely due to their ease of collection. Previous studies have found that the use of measures such as first arrival date reflect the widely-observed advancement of spring migration [112] and phenological first (and last) measures remain frequently-used, particularly so for migration studies assessing standardised datasets (e.g. [113], [114], [115], [116]).

In conclusion, the current evidence of climate change impacts on phenological patterns for many regions in the southern hemisphere is sparse, especially in South America, small island nations and Africa. Research efforts and policies are urgently needed to develop longer-term time series and more in-depth analyses. Despite locating more than 1000 datasets reviewed here, our understanding and capacity to predict phenological responses remains patchy and constrained. For a few species and communities we can identify some patterns, but we generally are unable to describe spatial (extents of change) and temporal (rates of change) responses. While we can make broad predictions and provide preliminary generalisations, our constrained understanding of the complexity of ecosystem relationships, structures and functions, and our incomplete knowledge on the spatial and temporal variabilities inherent in these ecosystems prevents more detailed analyses and predictions. While this collation has provided a baseline for future work, and has suggested many interesting avenues of enquiry, a vastly greater research effort is required to refine our understanding of the impacts of climate change on the phenologies of southern hemisphere species.

## Supporting Information

Appendix S1 **Long-term phenological data sets (>10 years in length) ending before 1990.** These were not used in the present study but could provide useful baseline data for future studies.
(PDF)

Appendix S2 **WinBUGS code used to estimate mean trends in phenology across plants and birds together with a plot of the posterior distribution for means and 95% credible intervals.** We used non-informative priors N(0, 10^6^) for the means and U(0, 100) for the standard errors, and ran three chains of length 1.2 million of which the first 200000 samples were discarded as burn-in and inference was drawn from the rest of the chains after thinning by a factor of 20. The model converged according to the R-hat statistic which was below 1.01 for all parameters, and we also visually inspected the chains.
(PDF)

Appendix S3 **Papers with long-term (at least 10 years) phenological data used in the analyses.**
(PDF)

Appendix S4 **Assessing the impact of the length of the data series on the likelihood of detecting a significant trend towards earlier or later phenologies.**
(PDF)

Appendix S5 **Breakdown of number of observations by family and species, based on the full data set (1208 time series).**
(PDF)

Appendix S6 **Summary of datasets and their trends using details of the phenophase.**
(PDF)
