# Supplementary material for: Phenological Changes in the Southern Hemisphere
Source: PLoS One. 2013 Oct 1;8(10):e75514. doi: 10.1371/journal.pone.0075514 (PMC3787957; doi:10.1371/journal.pone.0075514)
Supplement: Appendix S1 — Long-term phenological data sets (>10 years in length) ending before 1990. These were not used in the present study but could provide useful baseline data for future studies. (PDF) [file pone.0075514.s001.pdf]

Appendix S1 Long-term phenological data sets (>10 years in length) ending before 1990. These were not used in the present study but could provide useful baseline data for future studies.

Country	Taxa	No. of species	Years	Phenophases	Reference
Australia	Plant	49	1864-1885	Flowering, leafing, fruiting, harvest	Chambers & Keatley 2010
Australia	Plant	4	1940-1962	Flowering	Keatley et al. 2002
Australia	Aves	1	1975-1986	Laying dates	Olsen & Olsen 1989
New Zealand	Plant	1	1965-1988	Seed fall dates (monthly)	Allen & Platt 1990
New Zealand	Plant	7	1970-1989?	Flowering	Atkins & Morgan 1990
New Zealand	Aves	1	1943-1953	Arrival dates	Cunningham 1953
New Zealand	Aves	1	1970-1986	Laying dates	Flux 1987
New Zealand	Aves	1	1971-1982	Laying, fledging dates	Imber 1987
New Zealand	Aves	1	1936-1954	Laying dates	Richdale 1957
New Zealand	Aves	1	1954-1964	Laying, hatching dates	Williams 1967
Antarctica	Aves	1	1962-1975	Arrival, laying dates	Ainley et al. 1983
Sub-Antarctic	Mammalia	1	1950-1959	Peak haul out dates	Hindell & Burton 1988
Sub-Antarctic	Mammalia	1	1949-1979	First, last sighting	Rounsevell & Eberhard 1980

Ainley D, Russell J, Jenouvrier S, Woehler E, Lyver POB, Fraser WR, *et al.* (2010) Antarctic penguin response to habitat change as Earth's troposphere reaches 2° C above preindustrial levels. Ecol Monogr 80: 49-66.

Allen RB, Platt K (1990) Annual seedfall variation in *Nothofagus solandri* (Fagaceae), Canterbury, New Zealand. Oikos 57: 199-206.

Atkins T, Morgan E (1989) Modelling the effects of possible climate change scenarios on the phenology of New Zealand fruit crops. II International Symposium on Computer Modelling in Fruit Research and Orchard Management.

Chambers LE, Keatley MR (2010) Phenology and climate - early Australian botanical records. Aust J Bot 58: 473-484.

Cunningham JM (1953) The dates of arrival of the Shining-bronze Cuckoo in New Zealand in 1952. Notornis 5: 192-195.

Flux JEC (1987) Drift in laying dates of starlings *Sturnus vulgaris*. Ornis Scand 18: 146-148.

Hindell MA, Burton HR (1988) Seasonal haul-out patterns of the southern elephant seal (*Mirounga leonina* L.), at Macquarie Island. Journal of Mammalogy 69: 81-88.

Imber M (1987) Breeding ecology and conservation of the black petrel (*Procellaria parkinsoni*). Notornis 34: 19-39.

Keatley MR, Fletcher TD, Hudson IL, Ades PK (2002) Phenological studies in Australia: potential application in historical and future climate analysis. Int J Clim 22: 1769-1780.

Olsen P, Olsen J (1989) Breeding of the Peregrine Falcon *Falco peregrinus*. II. Weather, Nest Quality and the Timing of Egg Laying. Emu 89: 1-5.

Rounsevell D, Eberhard I (1980) Leopard Seals, *Hyrurga leptonyx* (Pinnipedia), at Macquarie Island from 1949 to 1979. Wildl Res 7: 403-415.

Williams G (1967) The breeding biology of California quail in New Zealand. Proc N Z Ecol Soc 14: 88-99.
