# Supplementary material for: Phenological Changes in the Southern Hemisphere
Source: PLoS One. 2013 Oct 1;8(10):e75514. doi: 10.1371/journal.pone.0075514 (PMC3787957; doi:10.1371/journal.pone.0075514)
Supplement: Appendix S2 — WinBUGS code used to estimate mean trends in phenology across plants and birds together with a plot of the posterior distribution for means and 95% credible intervals. We used non-informative priors N(0, 106) for the means and U(0, 100) for the standard errors, and ran three chains of length 1.2 million of which the first 200000 samples were discarded as burn-in and inference was drawn from the rest of the chains after thinning by a factor of 20. The model converged according to the R-hat statistic which was below 1.01 for all parameters, and we also visually inspected the chains. (PDF) [file pone.0075514.s002.pdf]

Appendix S2. WinBUGS code used to estimate mean trends in phenology across plants and birds together with a plot of the posterior distribution for means and 95% credible intervals. We used non-informative priors N(0, 10^6) for the means and U(0, 100) for the standard errors, and ran three chains of length 1.2 million of which the first 200000 samples were discarded as burn-in and inference was drawn from the rest of the chains after thinning by a factor of 20. The model converged according to the R-hat statistic which was below 1.01 for all parameters, and we also visually inspected the chains.

```
model {

# The data are mean trends per year for each time series ('trend.estimate')
#        and their standar errors ('se.estimate')
# Plants: phylum = 1; birds: phylum = 0
# The parameter 'beta' measures the difference between plants and birds
# time.spand is the logarithm of the length of the time series in years, rescaled to mean = 0
and unit sd
# beta.ts is the effect of time span on phenology trend

# Priors
mu.birds ~ dnorm(0, 0.0000001)          # mean trend for birds
beta ~ dnorm(0, 0.0000001)          # difference in trend between plants and birds
beta.ts ~ dnorm(0, 0.0000001)       # coefficient for time span
tau.series <- pow(sd.series, -2)
sd.series ~ dunif(0, 100)       # Among series heterogeneity
tau.species <- pow(sd.species, -2)
sd.species ~ dunif(0,100)

# Likelihood
for (i in 1:nspecies)  # for each of the n species
  {
   spp[i] ~ dnorm(0, tau.species)  # random species effect
  }
for (i in 1:n){
  trend.estimate[i] ~ dnorm(mu[i], tau.error[i])
  mu[i] ~ dnorm(mu.a[i], tau.series)
  mu.a[i] <- mu.birds + beta * phylum[i] + beta.ts * time.span[i] + spp[species[i]]
  tau.error[i] <- pow(se.estimate[i], -2)
}

# Derived quantities
mu.plants <- mu.birds + beta         # mean trend for plants

} # end model
```

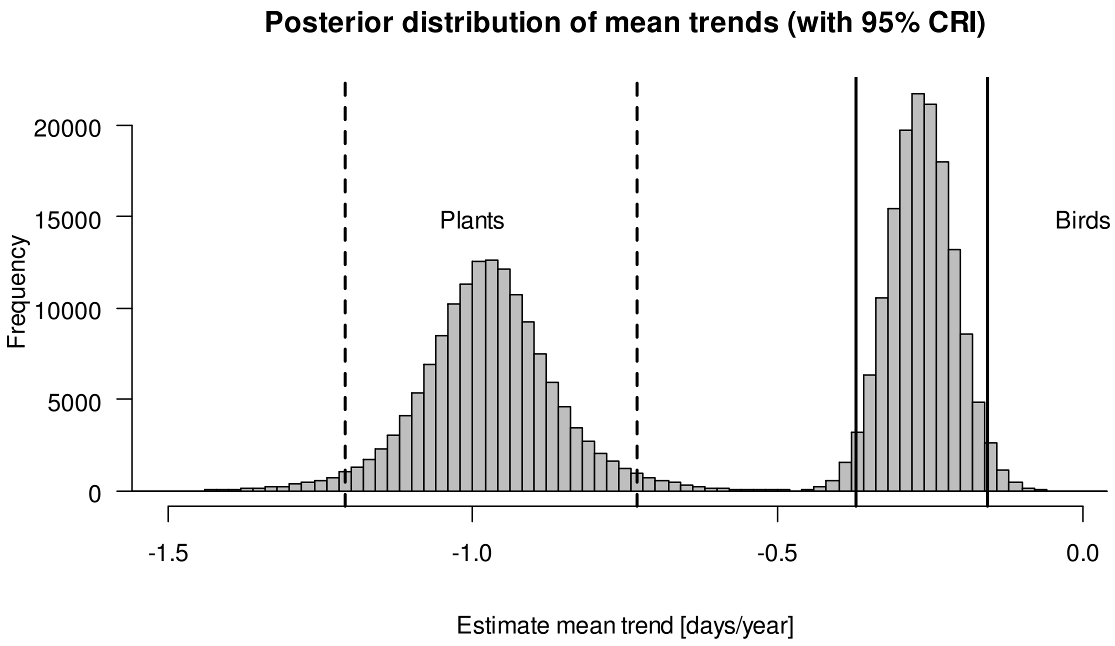
Posterior distribution of mean trends (with 95% CRI)
Frequency
20000
15000
10000
5000
0
Plants
Birds
-1.5
-1.0
-0.5
0.0
Estimate mean trend [days/year]
