# Supplementary material for: Phenological Changes in the Southern Hemisphere
Source: PLoS One. 2013 Oct 1;8(10):e75514. doi: 10.1371/journal.pone.0075514 (PMC3787957; doi:10.1371/journal.pone.0075514)
Supplement: Appendix S3 — Papers with long-term (at least 10 years) phenological data used in the analyses. (PDF) [file pone.0075514.s003.pdf]

Appendix S3. Papers with long-term (at least 10 years) phenological data used in the analyses.

Alencar JC (1994) Fenologia de cinco espécies arbóreas tropicais de Sapotaceae correlacionada a variáveis climáticas na Reserva Ducke, Manus, AM. *Acta Amazon*, 24, 161-182.

Alencar JC, Almeida RA,Fernandes N (1979) Fenologia de espécies florestais em floresta tropical úmida de terra firme na Amazônia Central. *Acta Amazon*, 9, 163-198.

Allen RB, Mason NWH, Richardson SJ, Platt KH (2011a) Synchronicity, periodicity and bimodality in inter-annual tree seed production along an elevation gradient. *Oikos*, 121, 367-376.

Allen WJ, Helps FW, Molles LE (2011b) Factors affecting breeding success of the Flea Bay white-flippered penguin (*Eudyptula minor albosignata*) colony. *N Z J Ecol*, 35, 199-208.

Altwegg R, Broms K, Erni B, Barnard P, Midgley GF, Underhill LG (2012) Novel methods reveal shifts in migration phenology of barn swallows in South Africa. *Proc R Soc B*, 279, 1485-1490.

Armstrong DP, Davidson RS, Dimond WJ, Perrott JK, Castro I, Ewen JG*, et al.* (2002) Population dynamics of reintroduced forest birds on New Zealand islands. *J Biogeogr*, 29, 609-621.

Barbraud C, Weimerskirch H (2006) Antarctic birds breed later in response to climate change. *PNAS*, 103, 6248-6251.

Barlow ML, Dowding JE (2002) Breeding biology of Caspian terns (*Sterna caspia*) at a colony near Invercargill, New Zealand. *Notornis*, 49, 76-90.

Baylis AMM, Zuur AF, Brickle P, Pistorius PA (2012) Climate as a driver of population variability in breeding Gentoo Penguins *Pygoscelis papua* at the Falkland Islands. *Ibis*, 154, 30-41.

Beaumont LJ, McAllan I, Hughes L (2006) A matter of timing: changes in arrival and departure dates of Australian migratory birds. *Glob Change Biol*, 12, 1339-1354.

Boersma PD, Rebstock GA (2009) Intraclutch egg-size dimorphism in Magellanic Penguins (*Spheniscus* magellanicus): adaptation, constraint, or noise? *The Auk,* 126, 335-340.

Bull CM, Burzacott D (2006) Changes in climate and in the timing of pairing of the Australian lizard, *Tiliqua rugosa*: a 15-year study. *J. Zool*, 256, 383-387.

Cannell B, Chambers L, Wooller R, Bradley S (2012) Poorer breeding by little penguins near Perth, Western Australia is correlated with above average sea surface temperatures and a stronger Leeuwin Current. *Mar Freshwat Res*, on-line first.

Chambers LE (2005) Migration dates at Eyre Bird Observatory: links with climate change? *Clim Res*, 29, 157-165.

Chambers LE (2008) Trends in timing of migration of south-western Australian birds and their relationship to climate. *Emu*, 108, 1-14.

Chambers LE (2010) Altered timing of avian movements in a peri-urban environment and its relationship to climate. *Emu*, 110, 48-53.

Chambers LE, Gibbs H, Weston MA, Ehmke GC (2008) Spatial and temporal variation in the breeding of Masked Lapwings (*Vanellus miles*) in Australia. *Emu*, 108, 115-124.

Chambers LE, Beaumont LJ, Hudson IL (*accepted*) Continental scale analysis of bird migration timing: influences of climate and life history traits - a generalised mixture model clustering and discriminant approach. *International Journal of Biometeorology.*

Crawford R, Dyer B, Cooper J, Underhill L (2006) Breeding numbers and success of Eudyptes penguins at Marion Island, and the influence of mass and time of arrival of adults. *CCAMLR Science*, 13, 175-190.

Cullen J, Chambers L, Coutin P, Dann P (2009) Predicting onset and success of breeding in little penguins *Eudyptula minor* from ocean temperatures. *Mar Ecol Prog Ser*, 378, 269-278.

Darbyshire R, Webb L, Goodwin I, Barlow EWR (*in press*) Evaluation of recent trends in Australian pome fruit spring phenology. *Int J Biometeorol*, DOI 10.1007/s00484-012-0567-1

de Morton J, Bye J, Pezza A, Newbigin E (2011) On the causes of variability in amounts of airborne grass pollen in Melbourne, Australia. *Int J Biometeorol*, 55, 613-622.

Dunlop J, Surman C (2012) The role of foraging ecology in the contrasting responses of two dark terns to a changing ocean climate. *Mar Ornithol*, 40, 105-110.

Durant JM, Crawford RJM, Wolfaardt AC, Agenbag K, Visagie J, Upfold L, *et al.* (2010) Influence of feeding conditions on breeding of African penguins- importance of adequate local food supplies. *Mar Ecol Prog Ser*, 420, 263-271.

Emmerson L, Pike R, Southwell C (2011) Reproductive consequences of environment-driven variation in Adélie penguin breeding phenology. *Mar Ecol Prog Ser*, 440, 203-216.

Engel VL, Martins FR (2005) Reproductive phenology of Atlantic forest tree species in Brazil: an eleven year study. *Trop Ecol*, 46, 1-16.

Evans KL, Tyler C, Blackburn TM, Duncan RP (2003) Changes in the breeding biology of the Welcome Swallow (*Hirundo tahitica*) in New Zealand since colonisation. *Emu*, 103, 215-220.

Forcada J, Trathan P, Reid K, Murphy E (2005) The effects of global climate variability in pup production of Antarctic fur seals. *Ecology*, 86, 2408-2417.

Fortescue M (1998) *The marine and terrestrial ecology of a northern population of the Little Penguin, Eudyptula minor, from Bowen Island, Jervis Bay*. PhD Thesis. University of Canberra.

Gallagher RV, Hughes L, Leishman MR (2009) Phenological trends among Australian alpine species: using herbarium records to identify climate-change indicators. *Aust J Bot*, 57, 1-9.

Gibbs H (2007) Climatic variation and breeding in the Australian Magpie (*Gymnorhina tibicen*): a case study using existing data. *Emu*, 107, 284-293.

Gibbs H, Chambers LE, Bennett AF (2011) Temporal and spatial variability in breeding in Australian birds: potential implications for climate change. *Emu*, 111, 283-291.

Grab S, Craparo A (2011) Advance of apple and pear tree full bloom dates in response to climate change in the southwestern Cape, South Africa: 1973–2009. *Agric For Meteorol*, 151, 406-413.

Green K (2010) Alpine taxa exhibit differing responses to climate warming in the Snowy Mountains of Australia. *J Mount Sci*, 7, 167-175.

Harris W, Beever RE, Parkes S, Webster R, Scheele S (2003) Genotypic variation of height growth and trunk diameter of *Cordyline australis* (Lomandraceae) grown at three locations in New Zealand. *N Z J Bot*, 41, 637-653.

Hindell MA, Bradshaw CJA, Brook BW, Fordham DA, Kerry K, Hull C, *et al.* (2012) Long-term breeding phenology shift in royal penguins. *Ecol Evol*, 2, 1563-1571.

Ifiiguez MA (2001) Seasonal distribution of killer whales (*Orcinus orca*) in Northern Patagonia, Argentina. *Aquatic Mammals*, 27, 154-l6l.

Imber M, West JA, Cooper WJ (2003) Cook's petrel (*Pterodroma cookii*): historic distribution, breeding biology and effects of predators. *Notornis*, 50, 221-230.

Kearney MR, Briscoe NJ, Karoly DJ, Porter WP, Norgate M, Sunnucks P (2010) Early emergence in a butterfly causally linked to anthropogenic warming. *Biol Lett*, 6, 674-677.

Keatley MR, Fletcher TD, Hudson IL, Ades PK (2004) *Shifts in the date of flowering commencement in some Australian plants.*16th Biometeorology and Aerobiology Conference. Vancouver, Canada. International Society of Biometeorology

Keatley MR, Hudson IL (2007) Shift in flowering dates of Australian plants related to climate: 1983–2006. In: *MODSIM 2007 International Congress on Modelling and Simulation. Land, Water and Environmental Management:* . Integrated Systems for Sustainability Modelling and Simulation Society of Australia and New Zealand, pp. 504-10.

Kelly D, Turnbull MH, Pharis RP, Sarfati MS (2008) Mast seeding, predator satiation, and temperature cues in Chionochloa (Poaceae). *Population Ecology*, 50, 343-355.

Khurshid T, Treeby M, Sanderson G (2010) *The effects of climatic extremes on phenological stages of navel oranges.*2010 International Climate Change Adaptation Conference. Gold Coast, Australia

Lescroël A, Bajzak C, Bost C-A (2009) Breeding ecology of the gentoo penguin Pygoscelis papua at Kerguelen Archipelago. *Polar Biol*, 32, 1495-1505.

Lima Jr MJ, Alencar JC (1992) *Fenologia de duas espécies do gênero Corythophora da família Lecythidaceae na Reserva Florestal Ducke, Manaus-AM.*2o Congresso Nacional sobre Essências Nativas.

Lynch H, Fagan W, Naveen R, Trivelpiece S, Trivelpiece W (2009) Timing of clutch initiation in Pygoscelis penguins on the Antarctic Peninsula: towards an improved understanding of off-peak census correction factors. *CCAMLR Sci*, 16, 149-165.

Lynch HJ, Fagan WF, Naveen R, Trivelpiece SG, Trivelpiece WZ (2012) Differential advancement of breeding phenology in response to climate may alter staggered breeding among sympatric pygoscelid penguins. *Mar Ecol Prog Ser*, 454, 135-145.

MacGillivrary F, Hudson IL, Lowe AJ (2010) Herbarium collections and photographic images: alternative data sources for phenological research. In: *Phenological Research* (eds. Hudson I.L. & Keatley M.R.). Springer, pp. 425-461.

Magalhães LMS, Alencar JC (1979) Fenologia do pau-rosa (*Aniba duckei* Kostermans), Lauraceae, em floresta primária da Amazônia Central. *Acta Amazon*, 9, 227-232.

McClellan K (2011) *The Responses of Australian Butterflies to Climate Change*. PhD Thesis. Department of Biological Sciences, Macquarie University.

McMahon C, Hindell MA (2009) Royal penguin phenology: changes in the timing of egg-laying of a Sub-Antarctic predator in response to a changing marine environment. In: *Seabird Group 10th International Conference VLIZ Special Publication 42. Communications of the Research Institute for Nature and Forest* (eds. Stienin E., Ratcliffe N., Seys J., Jürgen T., Mees J. & Dobbelaere I.). Research Institute for Nature and Forest (INBM), Brussels, Belgium - Flanders Marine Institute (VLIZ) Oostende, Belgium, p. 45.

Mills JA, Yarrall JW, Bradford-Grieve JM, Uddstrom MJ, Renwick JA, Merilä J (2008) The impact of climate fluctuation on food availability and reproductive performance of the planktivorous red-billed gull *Larus novaehollandiae scopulinus. J Anim Ecol*, 77, 1129-1142..

Møller AP, Nuttall R, Piper SE, Szép T, Vickers EJ (2011) Migration, moult and climate change in barn swallows Hirundo rustics in South Africa. *Clim Res*, 47, 201-205.

Norment C, Green K (2005) Breeding ecology of Richard's Pipit (*Anthus novaeseelandiae*) in the Snowy Mountains. *Emu*, 104, 327-336.

O'Donnell CFJ (2011) Breeding of the Australasian Bittern (*Botaurus poiciloptilus*) in New Zealand. *Emu*, 111, 197-201.

Peacock L, Paulin M, Darby J (2000) Investigations into climate influence on population dynamics of yellow-eyed penguins *Megadyptes antipodes*. *N Z J Zool*, 27, 317-325.

Petrie PR, Sadras VO (2008) Advancement of grapevine maturity in Australia between 1993 and 2006: putative causes, magnitude of trends and viticultural consequences. *Aust J Grape Wine Res*, 14, 33-45.

Pezzo F, Olmastroni S, Volpi V, Focardi S (2007) Annual variation in reproductive parameters of Adélie penguins at Edmonson Point, Victoria Land, Antarctica. *Polar Biol*, 31, 39-45.

Pinto AM, Morellato L, Barbosa AP (2008) Reproductive phenology of *Dipteryx odorata* (Aubl.) Willd (Fabaceae) in two forest areas in the Central Amazon. *Acta Amazon*, 38, 643-649.

Pinto AM, Ribeiro RJ, Alencar JC, Barbosa AP (2005) Fenologia de *Simarouba amara* Aubl. na Reserva Florestal Adolpho Ducke, Manaus, AM. *Acta Amazon*, 35, 643-649.

Pye D, Dowding J (2002) Nesting period of the northern New Zealand dotterel (*Chradrius obscurus aquilonius*). *Notornis*, 49, 259-260.

Robertson C (1993) Survival and longevity of the Northern Royal Albatross *Diomedea epomophora sanfordi* at Taiaroa Head 1937-93. *Emu*, 93, 269-276.

Rosas F, Pinedo M, Marmotel M, Haimovici M (1994) Seasonal movements of the South American sea lion (*Otaria flavescens* Shaw) off the Rio Grande do Sul coast, Brazil. *Mammalia*, 58, 51-60.

Ruiz J, Alencar J (1999) Phenological interpretation of five Chrysobalanaceae tree species in the Adolpho Ducke Forest Reserve, Manaus, Amazonas, Brazil. *Acta Amazon*, 29, 223-242.

Ruiz RR, Alencar JC (2004) Comportamento fenológico da palmeira patauá (*Oenocarpus bataua*) na reserva florestal Adolpho Ducke, Manaus, Amazonas, Brasil. *Acta Amazon*, 34, 553-558.

Rumpff L, Coates F, Messina A, Morgan J (2008) Potential biological indicators of climate change: evidence from phenology records of plants along the Victorian coast. In. Arthur Rylah Institute for Environmental Research Technical Report No. 175, Victorian Department of Sustainability and Environment.

Rumpff L, Coates F, Morgan JW (2010) Biological indicators of climate change: evidence from long-term flowering records of plants along the Victorian coast, Australia. *Aust J Bot*, 58, 428-439.

Sadras, V. & Petrie, P. (2011). Climate shifts in south-eastern Australia: early maturity of Chardonnay, Shiraz and Cabernet Sauvignon is associated with early onset rather than faster ripening. *Aust J Grape Wine Res*, 17, 199-205.

Sagar P, Geddes D, Banks JC, Howden P (2000) Breeding of South Island pied oystercatchers (*Haematopus ostralegus finschi*) on farm land in mid-Canterbury, New Zealand. *Notornis*, 47, 71-81.

Sagar P, Miskelly C, Sagar J, Tennyson AJD (2003) Population size, breeding, and annual cycle of the New Zealand Antarctic tern (*Sterna vittata bethunei*) at the Snares Islands. *Notornis*, 50, 36-42.

Saraux C, Le Bohec C, Durant JM, Viblanc VA, Gauthier-Clerc M, Beaune D*, et al.* (2011) Reliability of flipper-banded penguins as indicators of climate change. *Nature*, 469, 203-206.

Saul EK, Robertson HA, Tiraa A (1998) Breeding biology of the kakerori (*Pomarea dimidiata*) on Rarotonga, Cook Islands. *Notornis*, 45, 255-268.

Schloss IR, Abele D, Moreau S, Demers S, Bers AV, González O, *et al.* (2011) Response of phytoplankton dynamics to 19-year (1991–2009) climate trends in Potter Cove (Antarctica). *Journal of Marine Systems*, 92, 53-66.

Senapathi D, Nicoll MAC, Teplitsky C, Jones CG, Norris K (2011) Climate change and the risks associated with delayed breeding in a tropical wild bird population. *Proc R Soc Lond Ser B Biol Sci*, 278, 3184-3190.

Slip DJ, Burton HR (1999) Population status and seasonal haulout patterns of the southern elephant seal (*Mirounga leonina*) at Heard Island. *Antarct Sci*, 11, 38-47.

Smith P, Smith J (2012) Climate change and bird migration in south-eastern Australia. *Emu*, 112, 333-342.

Surman CA, Nicholson LW (2009a) El Niño Southern Oscillation and the Leeuwin Current influence on seabird reproductive performance and diet at the Houtman Abrolhos. *Journal of the Royal Society of Western Australia*, 92, 155-163.

Surman CA, Nicholson LW (2009b) The good, bad and the ugly: ENSO driven oceanographic variability and its influence on seabird diet and reproductive performance at the Houtman Abrolhos, eastern Indian Ocean. *Mar Ornithol*, 37, 129-138.

Surman CA, Nicholson LW, Santora JA (2012) Effects of climate variability on breeding phenology and performance of tropical seabirds in the eastern Indian Ocean. *Mar Ecol Prog Ser*, 454, 147-157.

Telemeco RS, Elphick MJ, Shine R (2009) Nesting lizards (*Bassiana duperreyi*) compensate partly, but not completely, for climate change. *Ecology*, 90, 17-22.

Tryjanowski P, Flux JEC, Sparks TH (2006) Date of breeding of the starling *Sturnus vulgaris* in New Zealand is related to El Niño Southern Oscillation. *Austral Ecol*, 31, 634-637.

Underhill L, Crawford R (1999) Season of moult of African penguins at Robben Island, South Africa, and its variation, 1988–1998. *S Afr J Mar Sci*, 21, 437-441.

Webb L, Whetton P, Barlow E (2011) Observed trends in winegrape maturity in Australia. *Glob Change Biol*, 17, 2707-2719.

Webb L, Whetton P, Bhend J, Darbyshire R, Briggs P, Barlow E (2012) Earlier wine-grape ripening driven by climatic warming and drying and management practices. *Nature Clim Change*, 2, 259-264.

Wolfaardt A, Underhill L, Crawford R (2009a) Comparison of moult phenology of African penguins *Spheniscus demersus* at Robben and Dassen islands. *Afr J Mar Sci*, 31, 19-29.

Wolfaardt A, Underhill L, Visagie J (2009b) Breeding and moult phenology of African penguins *Spheniscus demersus* at Dassen Island. *Afr J Mar Sci*, 31, 119-132.
