# Supplementary material for: Phenological Changes in the Southern Hemisphere
Source: PLoS One. 2013 Oct 1;8(10):e75514. doi: 10.1371/journal.pone.0075514 (PMC3787957; doi:10.1371/journal.pone.0075514)
Supplement: Appendix S4 — Assessing the impact of the length of the data series on the likelihood of detecting a significant trend towards earlier or later phenologies. (PDF) [file pone.0075514.s004.pdf]

Appendix S4. Assessing the impact of the length of the data series on the likelihood of detecting a significant trend towards earlier or later phenologies.

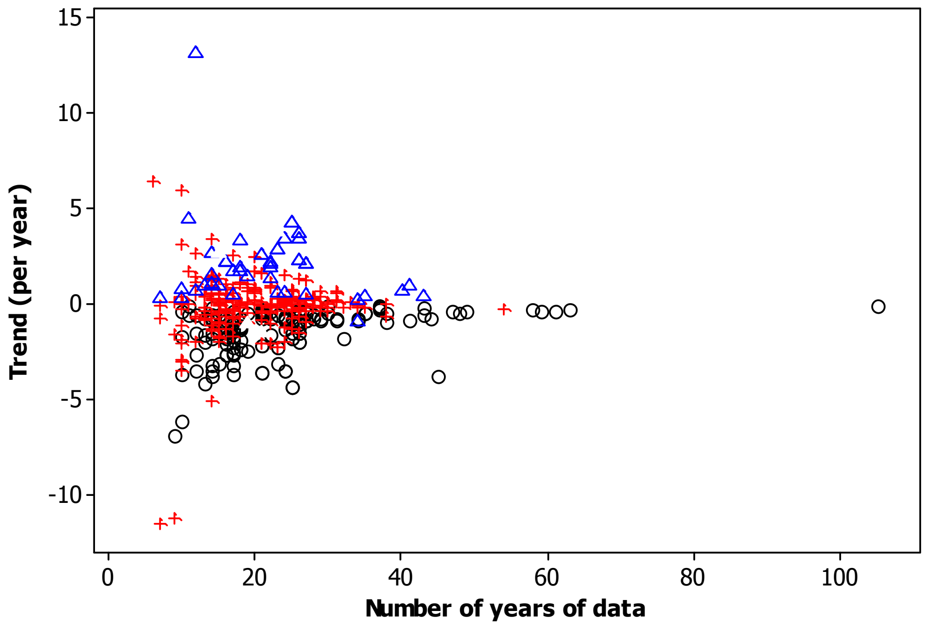

Figure 1. Strength of trend (days per year) versus number of years used to calculate the trend (triangles significantly earlier, circles significantly later, + non-significant trends).

There was no impact of the length of the data series or the year in which the data series commenced on the likelihood of detecting an earlier or later trend over time (Sections S4.1 and S4.2, respectively). However the length of the data series clearly influenced the magnitude of the trend observed (Figure 1; Section S4.3), with the start year having only a marginal effect. In general, the magnitude of the phenological trend was greater for shorter data series and for those that started more recently.

All analyses were conducted using the Minitab Statistical Software (Release 14, Minitab Inc., www.minitab.com)

S4.1 Ordinal Logistic Regression: Influence of number of years of data on significance of trend

Here we use ordinal logistic regression to perform a logistic regression on an ordinal response variable, representing the significance and direction of the phenological trend. This technique is designed to deal with ordinal variables, i.e. categorical variables that have three or more possible levels with a natural ordering, in our case earlier, no change and later. Iterative-reweighted least squares algorithm is used to obtain maximum likelihood estimates of the

parameters. The values labelled Const(1) and Const(2) are estimated intercepts for the logits of the cumulative probabilities of 'earlier', and for 'no change', respectively. Because the cumulative probability for the last response value is 1, there is not need to estimate an intercept for 'later'.

Link Function: Logit $g(\chi) = \log_e(\chi/(1-\chi))$

where χ is the cumulative probability up to and including the relevant category of the response.

Response Information:

Variable: SIGN, defined as -1 for significantly earlier trends, 1 for significantly later trends and 0 for non-significant trends.

Value	Count
-1 (Earlier)	157
0 (No change)	315
1 (Later)	66
Total	538

NOTE: 538 cases were used; 670 cases contained missing values

Logistic Regression Table:

Predictor	Coefficient	SE Coeff.	Z	P-value	Odds Ratio	95% CI
Const(1)	-1.2408	0.2120	-5.85	<0.001		
Const(2)	1.6261	0.2218	7.33	<0.001		
No. years of data	0.0163	0.0085	1.92	0.055	1.02	(1.00, 1.03)

Log-Likelihood = -498.759
Test that all slopes are zero: G = 3.402, DF = 1, P-Value = 0.065

Goodness-of-Fit Tests

Method	Chi-square	df	P-value
Pearson	136.90	93	0.002
Deviance	136.58	93	0.002

Measures of Association: (Between the Response Variable and Predicted Probabilities)

Pairs	Number	Percent	Summary Measures	
Concordant	40585	50.3	Somers' D	0.06
Discordant	35927	44.6	Goodman-Kruskal Gamma	0.06
Ties	4095	5.1	Kendall's Tau-a	0.03
Total	80607	100.0		

S4.2 Ordinal Logistic Regression: Influence of number of years of data and start year

See S4.1 for analysis details and variable definitions.

Link Function: Logit

Response Information: Variable: SIGN

Value	Count
-1 (Earlier)	156
0 (No change)	315
1 (Later)	66
Total	537

NOTE: 537 cases were used; 671 cases contained missing values

Logistic Regression Table

Predictor	Coefficient	SE Coeff.	Z	P-value	Odds Ratio	95% CI
Const(1)	-6.0351	12.1757	-0.50	0.620		
Const(2)	-3.1622	12.1727	-0.26	0.795		
No. years of data	0.0185	0.0095	1.95	0.052	1.02	(1.00, 1.04)
Start year	0.0024	0.0061	0.39	0.695	1.00	(0.99, 1.01)

Log-Likelihood = -497.323
Test that all slopes are zero: G = 3.805, DF = 2, P-Value = 0.149

Goodness-of-Fit Tests

Method	Chi-square	df	P-value
Pearson	629.172	530	0.002
Deviance	621.278	530	0.004

Measures of Association: (Between the Response Variable and Predicted Probabilities)

Pairs	Number	Percent	Summary Measures	
Concordant	41979	52.3	Somers' D	0.08
Discordant	35808	44.6	Goodman-Kruskal Gamma	0.08
Ties	2439	3.0	Kendall's Tau-a	0.04
Total	80226	100.0		

S4.3 Regression: Magnitude of trend and years of data

All trends (significant or not) were transformed to positive values only and natural logarithm taken. The data series of length 105 years is an outlier and was removed.

The regression equation is
Loge(TrendMagnitude) = 0.2239 - 0.03160 (No.Yrs of Data)

S = 1.27115   R-Sq = 4.2%   R-Sq(adj) = 4.0%

Analysis of Variance

Source	df	SS	MS	F	P-value
Regression	1	32.875	32.876	20.35	<0.001
Error	462	746.511	1.616		
Total	463	779.386			

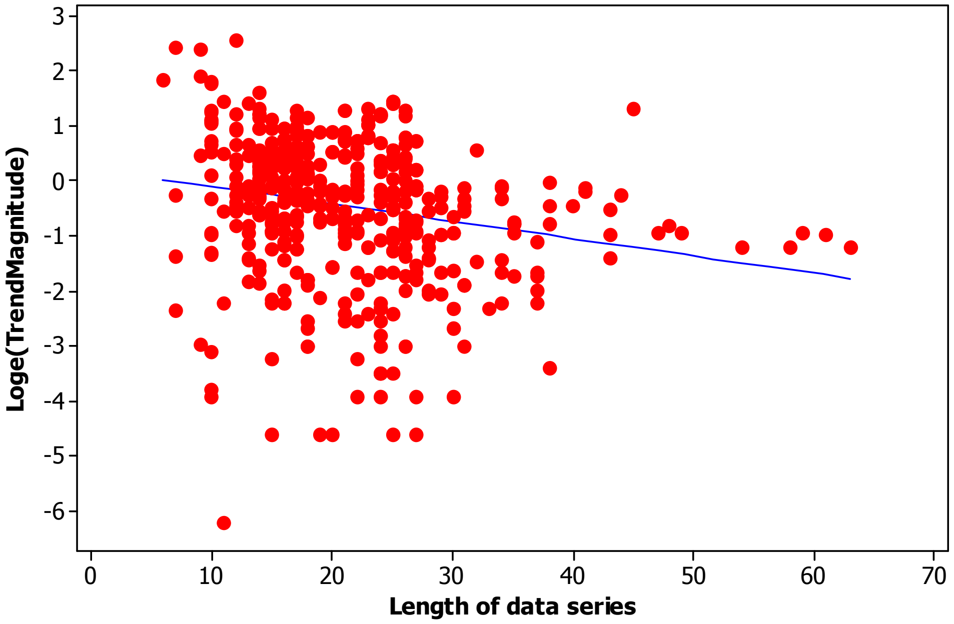

Including year the observations started:

The regression equation is
Loge(TrendMagnitude) = - 17.1 - 0.0270 (No. yrs data) + 0.00873 (Start yr)

463 cases used, 745 cases contain missing values

Predictor	Coefficient	SE Coef.	T	P-value
Constant	-17.107	8.281	-2.07	0.039
No. yrs data	-0.027	0.007	-3.63	<0.001
Start year	0.009	0.004	2.10	0.037

S = 1.26410   R-Sq = 5.3%   R-Sq(adj) = 4.9%
