# Supplementary material for: Phenological Changes in the Southern Hemisphere
Source: PLoS One. 2013 Oct 1;8(10):e75514. doi: 10.1371/journal.pone.0075514 (PMC3787957; doi:10.1371/journal.pone.0075514)
Supplement: Appendix S5 — Breakdown of number of observations by family and species, based on the full data set (1208 time series). (PDF) [file pone.0075514.s005.pdf]

Appendix S5. Breakdown of number of observations by family and species, based on the full data set (1208 times series).

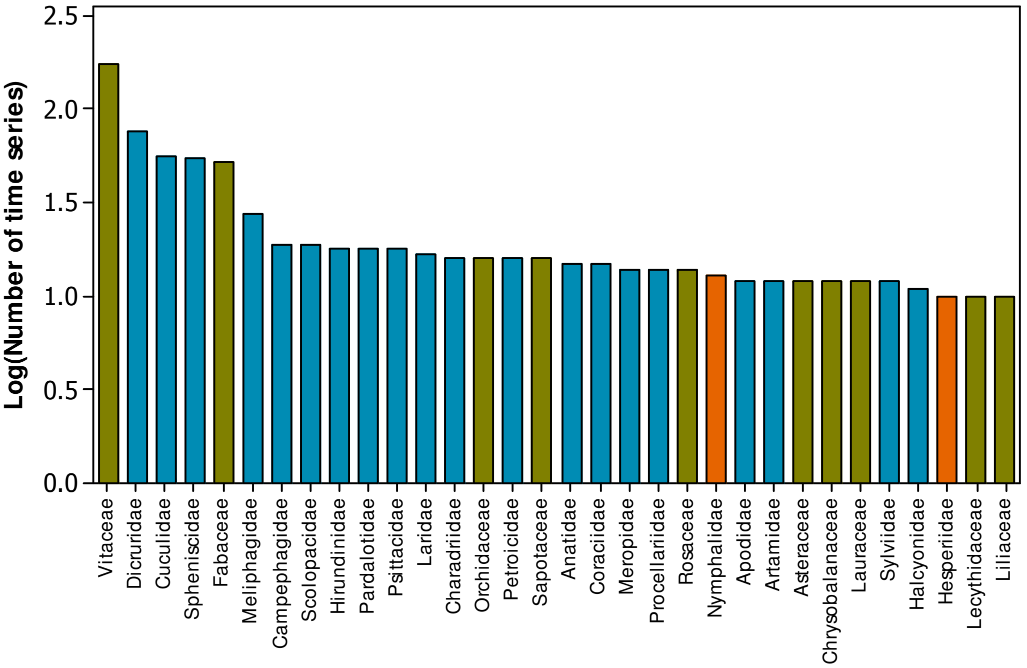

Figure S5.1. Number of observations for each family (restricted to those with at least 10 observations). Plant species represented by green bars, birds by blue bars and arthropods by orange bars.

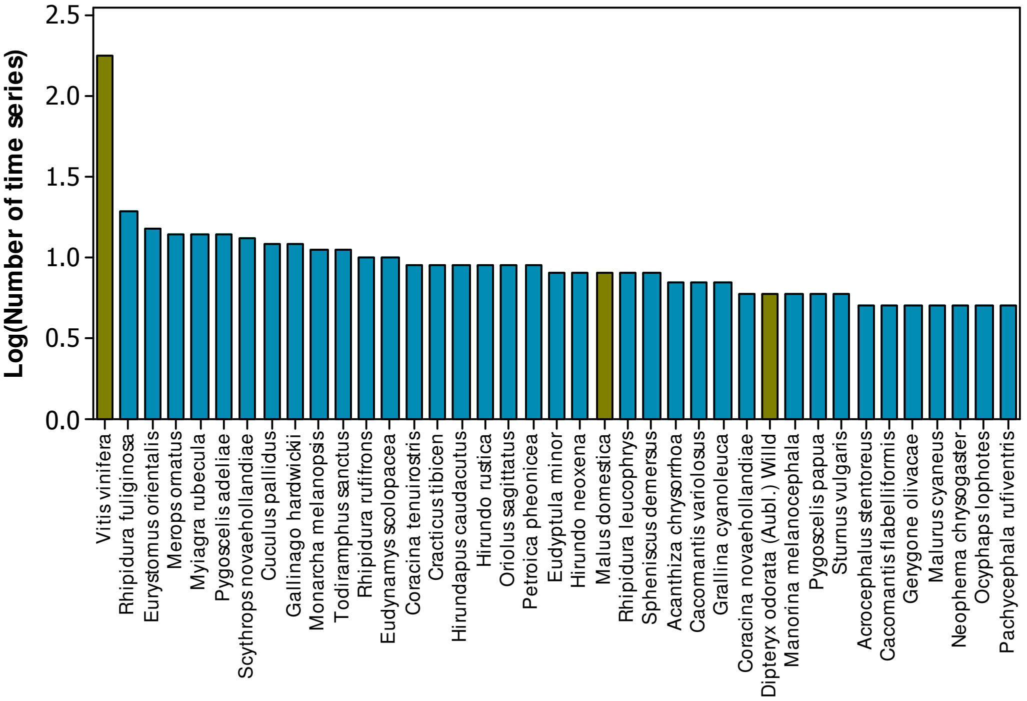

Figure S5.2. Number of observations for each species (restricted to those with at least 5 observations). Plant species represented by green bars and birds by blue bars.
