# Supplementary material for: Phenological Changes in the Southern Hemisphere
Source: PLoS One. 2013 Oct 1;8(10):e75514. doi: 10.1371/journal.pone.0075514 (PMC3787957; doi:10.1371/journal.pone.0075514)
Supplement: Appendix S6 — Summary of datasets and their trends using details of the phenophase. (PDF) [file pone.0075514.s006.pdf]

Appendix S6 Summary of datasets and their trends using details of the phenophase

Table S6.1. Breakdown of long-term phenological data sets (>10 years in length) by phenophase. Number of datasets covers all datasets that have the same type of phenophase observed. Mean trends are only calculated if at least 5 datasets in this category, S.E. is the standard error of the mean trend estimate and N is the number of datasets in this category that reported a trend estimate.

Taxa	Phenophase	Details of phenophase	Number of datasets	Mean trend (S.E.; N)
Arthropoda	Migration	First arrival date	2	-
	Emergence	First emergence date	1	-
		First flight date	157	0.243 (0.180; 25)
		First sighting date	1	-
Aves	Breeding	Age at first breeding	2	-
		First laying date	20	-1.693 (0.635; 5)
		First laying date & mean laying date	3	-
		Mean first laying date	1	-
		Mean laying date	13	- (-; 4)
		Median laying date	3	-
		Modal egg date	1	-
		Laying date	21	- (-; 3)
		Last laying date	3	-
		Timing of nesting	3	-
		Mean breeding timing	4	-
		Timing of breeding	1	-
		Incubation date	1	-
		Number of pairs incubating	1	-
		Hatching date	83	0.125 (0.101; 77)
		Creching date	1	-
		First chick banding date	1	-
		Mean chick banding date	1	-
		Fledging date	2	-
		Fledging departure date	2	-
		Mean fledging date	2	-
		Timing of fledging	1	-
		Season length	1	-
	Migration	Arrival date	3	-
		Arrival date: females	2	-
		Arrival date: males	2	-
		Arrival: proportion of checklist	3	-
		First arrival date	200	-0.305 (0.106; 179)
		Last departure date	131	0.061 (0.176; 120)

		Departure: proportion of checklist	3	-
		Departure: female	1	-
		Departure: male	1	-
		Peak abundance date	27	0.038 (0.755; 27)
		Season length	9	
	Moult	Moult peak date	3	-
Mammalia	Breeding	Birth date	1	-
	Haulout	Date maximum number adults	1	-
	Migration	Abundance timing	2	-
Phytoplankton	Biomass		1	-
Plant	Harvest	Date of designated maturity	74	-1.262 (0.108; 74)
		Harvest commenced	45	-1.285 (0.085; 45)
	Maturity	Date of designated maturity	45	-1.404 (0.089; 45)
		Duration linear increase in total	3	-
		Onset linear increase in total	3	-
		Threshold time for maximum conc.	3	-
		Time when berries reach 12 Be	3	-
	Flowering	Bloom date	4	-
		First flowering date	23	- (-; 1)
		Flowering commenced	69	-0.284 (0.455; 17)
		Flowering	5	-0.810 (0.144; 5)
		Full bloom	7	-0.091 (0.061; 7)
		Full flowering	1	-
		Peak flowering	3	-
		Peak % trees	85	- (-; 3)
		Years to first flower	1	-
		(unspecified)	11	
	Budding	Green tip	1	-
	Fruiting	Peak % trees	85	- (-; 0)
	Seeding	Mast seeding	4	-
		Seed fall	2	-
	Pollen	Season start	1	-
		Season end	1	-
		Season length	1	-
Reptilia	Breeding	End of pairing	1	-
		Start of pairing	1	-
		Timing of oviposition	1	-

Table S6.2. Statistics related to type of phenophase. Codes: F first date; L last date; M mean, median or peak date; O other definition of timing or insufficient detail provided in source paper to correctly identify. Mean trends are only calculated if at least 5 datasets in this category, S.E. is the standard error of the mean trend estimate.

Taxa	Phenophase	Phenophase Code	Number of datasets with trends reported	Mean trend (S.E.)
Arthropoda	Migration	F	1	-
	Emergence	F	26	0.228 (0.174)
Aves	Breeding	F	6	-1.453 (0.571)
		L	2	-
		M	11	-0.355 (0.241)
		O	81	0.111 (0.096)
	Migration	F	179	-0.305 (0.106)
		L	120	0.061 (0.176)
		M	27	0.038 (0.755)
		O	10	0.350 (0.516)
Plant	Harvest	F	45	-1.285 (0.085)
		O	74	-1.262 (0.108)
	Maturity	O	45	-1.404 (0.089)
	Flowering	F	18	-0.307 (0.429)
		M	11	-0.143 (0.077)
		O	9	-0.519 (0.138)
	Budding	O	1	-
	Pollen	F	1	-
		L	1	-
		O	1	-
Reptilia	Breeding	F	1	-
		L	1	-
